# In Vivo Extracellular Recording Reveals Bidirectional Changes in Neuronal Activity in the Rat Spinal Dorsal Horn After Hindlimb Ischemia–Reperfusion

**DOI:** 10.3390/ijms27104254

**Published:** 2026-05-10

**Authors:** Daisuke Uta, Keita Takeuchi, Kazuo Yano, Keigo Fukano, Tatsuro Minami, Akitoshi Ito

**Affiliations:** 1 Department of Applied Pharmacology, Faculty of Pharmaceutical Sciences, University of Toyama, Toyama 930-0194, Japan; 2 Laboratory for Pharmacology, Pharmaceutical Research Center, Asahi Kasei Pharma Corporation, Shizuoka 410–2321, Japan; takeuchi.kt@om.asahi-kasei.co.jp (K.T.); yano.kk@om.asahi-kasei.co.jp (K.Y.); fukano.kf@om.asahi-kasei.co.jp (K.F.); minami.tf@om.asahi-kasei.co.jp (T.M.); ito.ab@om.asahi-kasei.co.jp (A.I.)

**Keywords:** ischemia-reperfusion, neuropathy, dorsal horn, rat model, spontaneous firing, mRNA expression, behavioral analysis

## Abstract

Peripheral nerve ischemia–reperfusion injury is considered to contribute to sensory disturbances that impair quality of life in patients with diabetic neuropathy and chemotherapy-induced neuropathy. However, the spinal mechanisms underlying these disturbances remain unclear, partly due to the lack of established animal models and evaluation systems. In the present study, we used a rat hindlimb ischemia–reperfusion model and in vivo extracellular recording to examine bidirectional changes in neuronal activity in the spinal dorsal horn. Ischemia was induced by tightly binding the rat ankle with a rubber band, followed by reperfusion. Behavioral analysis showed a significant increase in hindlimb licking behavior after reperfusion, indicating the development of sensory disturbance-like responses. Extracellular recordings from superficial dorsal horn neurons showed diverse patterns of spontaneous firing and responses to mechanical stimulation, with both hypersensitive and desensitized responses. Furthermore, mRNA expression levels of immediate early genes (*Egr1*, *Egr3*, and *Fos*) were upregulated in the spinal cord after reperfusion. These results suggest that this ischemia–reperfusion model reproduces complex neuronal responses relevant to peripheral neuropathy and provides a useful evaluation system for evaluating both increased and decreased neural activity. This approach may contribute to elucidating the mechanisms of sensory disturbances and to the development of new treatments for neuropathic conditions.

## 1. Introduction

Sensory disturbances associated with diabetic neuropathy or chemotherapy-induced neuropathy are often unpleasant and intolerable, substantially impairing quality of life (QOL) [1,2]. Although these symptoms profoundly affect patients, the mechanisms underlying their transmission and regulation are still poorly understood, and therapeutic development has been constrained [3,4]. One major reason for this is the absence of validated animal models and standardized assessment methods for preclinical research.

Ischemia–reperfusion refers to the process in which tissue or organ damage occurs due to a temporary reduction in blood flow (ischemia) followed by a sudden restoration of blood flow (reperfusion), leading to inflammation and injury [5,6]. Peripheral nerves are also affected during this process [7,8], resulting in sensory disturbances due to disrupted nerve transmission. Transient ischemia–reperfusion, such as that caused by sitting with crossed legs, is known to induce tingling and other sensory symptoms. This results from reduced blood flow and oxygen supply to peripheral nerves caused by blood vessel compression, impairing nerve cell function and conduction. When blood flow resumes, abnormal electrical signals may be generated until ionic balance is restored, producing transient sensory symptoms [9].

Changes in peripheral blood flow are closely related to the occurrence of sensory disturbances. For example, in diabetic neuropathy [10,11,12] and chemotherapy-induced neuropathy [13,14,15], vascular damage caused by hyperglycemia or anticancer drugs such as oxaliplatin and paclitaxel leads to ischemia–reperfusion and subsequent peripheral nerve damage, which is believed to contribute to sensory symptoms.

Previously, we developed a mouse model of ischemia–reperfusion by tightly binding the ankle with a rubber band to reduce blood flow and then releasing it to restore circulation [9,16]. This model successfully reproduced sensory disturbances, as evidenced by increased licking behavior and spontaneous firing in peripheral nerves. Another group reported that mechanical stimulation of the hindlimb resulted in suppressed escape behavior, indicating reduced sensory function [17]. Since neuropathic sensory symptoms in humans include tingling, pricking, pins-and-needles, pain, and numbness (decrease or loss of sensation) [18,19], it is important to establish an evaluation system that can analyze both increased and decreased neural activity.

Sensory disturbances are often accompanied by pain. Spontaneous firing in the peripheral sensory nerves (such as the saphenous nerve) in the mouse ischemia–reperfusion model [9] suggests that sensory information is transmitted to the superficial dorsal horn of the spinal cord via primary sensory neurons. Painful sensory information is conveyed by myelinated Aδ and unmyelinated C fibers to the superficial layers (I, II) of the dorsal horn [20], where it is either directly transmitted to secondary neurons or modified by interneurons before transmission [21]. However, there have been no reports of altered sensory responses recorded from the dorsal horn.

We have also established in vivo extracellular recording from rat spinal dorsal horn neurons, enabling the measurement of spontaneous firing and responses to mechanical stimulation [22,23]. Therefore, the present study aimed to apply the mouse ischemia–reperfusion model to rats and use in vivo extracellular recording to capture the effects of hindlimb ischemia–reperfusion on neuronal activity in the rat spinal dorsal horn.

## 2. Results

### 2.1. Behavioral Changes and Plantar Blood Flow Induced by Ischemia–Reperfusion

**Figure 1:**
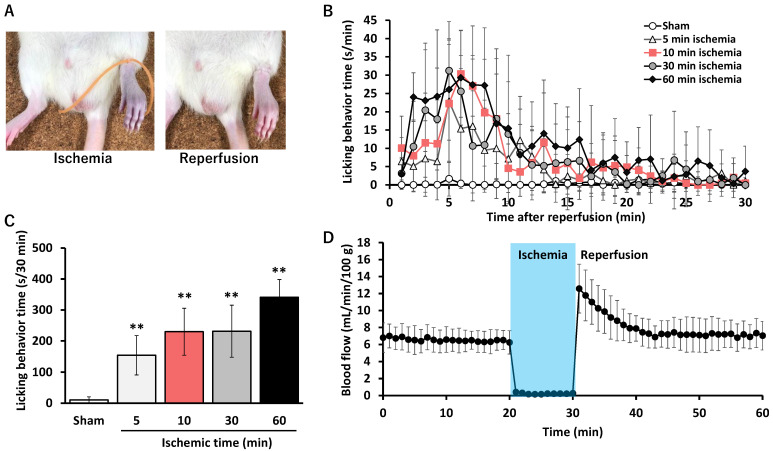
Licking behavior and changes in plantar blood flow after the ischemia–reperfusion procedure on the rat hindlimb. (**A**) Schematic of the ischemia–reperfusion procedure. The rat hindlimb is tightly bound with a rubber band for 5, 10, 30, or 60 min to induce ischemia, followed by reperfusion. (**B**) The time course of hind paw licking after 5, 10, 30, or 60 min of ischemia and subsequent reperfusion. Data are presented as mean ± standard deviation (*n* = 8 each). ** *p* < 0.01 vs. sham (ANOVA with post hoc Dunnett’s test). (**C**) The total licking time for each ischemia duration. Total licking was measured for 30 min after the ischemia–reperfusion procedure. Data are presented as mean ± standard deviation (*n* = 8 each). (**D**) Changes in plantar blood flow after 10 min of ischemia and subsequent reperfusion. Plantar blood flow was monitored using a laser Doppler flowmeter. Data are presented as mean ± standard deviation (*n* = 4).

To assess behavioral responses induced by hindlimb ischemia–reperfusion, ischemia was induced by tightly binding the rat ankle with a rubber band (Figure 1A), and licking behavior was measured following reperfusion. As shown in Figure 1B, rats subjected to ischemia for 5, 10, 30, or 60 min exhibited a significant increase in licking time compared with the control (Sham) group. The time course analysis showed that licking behavior peaked within the first 10 min after reperfusion and decreased gradually, but persisted throughout the 30 min observation period.

The longest licking time was observed after 60 min of ischemia (Figure 1C), although self-injurious behavior may have contributed to this increase. Considering that, at 60 min, licking behavior may be further increased due to wounds caused by self-injurious behavior, it is possible that the true licking behavior reaches a plateau at 10 min of ischemia. Therefore, in subsequent analyses, a 10 min ischemia duration was selected to examine neural responses.

Figure 1D illustrates the changes in plantar blood flow following ischemia–reperfusion. When the rat ankle was tightly bound with a rubber band to induce ischemia, plantar blood flow decreased rapidly and was almost completely blocked within one minute. After maintaining ischemia for 10 min, reperfusion resulted in a marked increase in blood flow, which then decreased gradually and returned to baseline within approximately 15 min.

### 2.2. Diversity of Neuronal Responses in the Spinal Dorsal Horn

**Figure 2:**
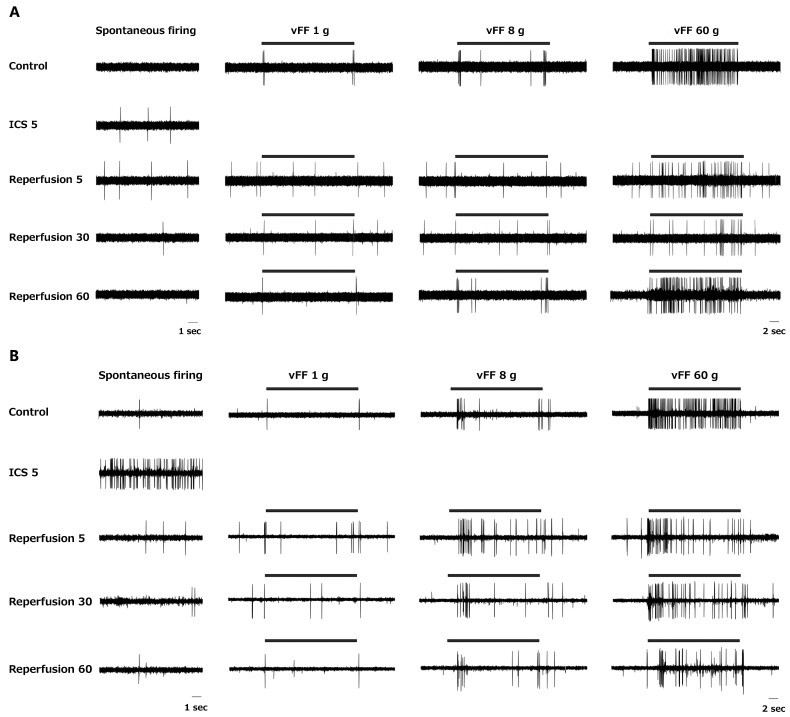
Representative extracellular recordings from superficial dorsal horn neurons in the rat spinal cord before, during, and after ischemia–reperfusion. Traces show spontaneous firing and responses to mechanical stimulation (von Frey filaments: 1 g, 8 g, and 60 g) recorded from neurons located in the superficial dorsal horn of the rat spinal cord under control conditions (before ischemia), 5 min (ICS5) after the onset of ischemia, and at 5, 30, and 60 min after reperfusion. (**A**) Example 1: Spontaneous firing increases during ischemia and, after reperfusion, recovers gradually between 30 and 60 min. The firing frequency in response to 1 g and 8 g stimuli does not change markedly after reperfusion, whereas firing in response to 60 g stimulation decreases after reperfusion and recovers by 60 min. (**B**) Example 2: Spontaneous firing increases markedly during ischemia and decreases immediately after reperfusion. The firing frequency in response to 1 g and 8 g stimuli increases after reperfusion and returns to baseline by 60 min. In contrast, firing in response to 60 g stimulation decreases after reperfusion and remains suppressed.

Representative extracellular recordings from superficial dorsal horn neurons demonstrated diverse patterns of spontaneous firing and responses to mechanical stimulation (von Frey filaments (vFFs) 1 g, 8 g, and 60 g) before and after ischemia–reperfusion. As shown in Figure 2, two distinct examples were observed.

In Example 1 (Figure 2A), spontaneous firing increased during ischemia and gradually recovered between 30 and 60 min after reperfusion. Firing frequency in response to 1 g and 8 g stimuli did not change markedly after reperfusion, whereas firing in response to 60 g stimulation decreased after reperfusion and recovered by 60 min.

In Example 2 (Figure 2B), spontaneous firing increased markedly during ischemia and decreased immediately after reperfusion. The firing frequency in response to 1 g and 8 g stimuli increased after reperfusion and returned to baseline by 60 min, whereas firing in response to 60 g stimulation decreased after reperfusion and remained suppressed.

These examples illustrate the diversity of neuronal responses to ischemia–reperfusion in the spinal dorsal horn.

### 2.3. Quantitative Analysis of Spontaneous Firing

**Figure 3:**
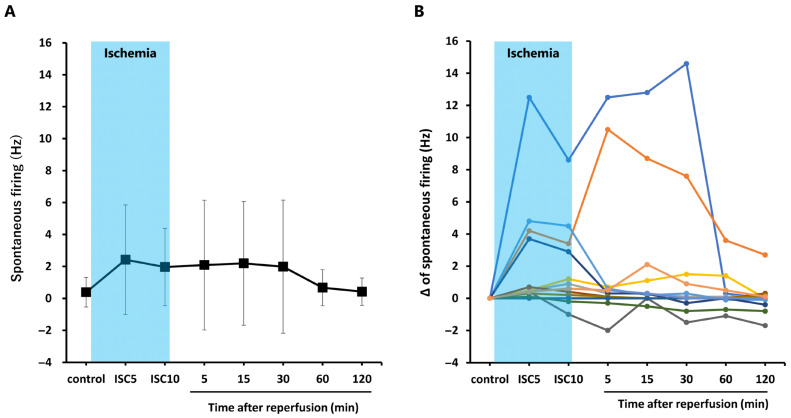
Changes in spontaneous firing of neurons in the superficial dorsal horn of the rat spinal cord during ischemia and after reperfusion. Spontaneous firing frequency (Hz) was measured at 5 min (ICS5) and 10 min (ICS10) after the onset of ischemia, as well as at 5, 15, 30, 60, and 120 min following reperfusion. (**A**) Changes in the average frequency of spontaneous firing. Data are presented as mean ± standard deviation (*n* = 14). (**B**) The change (Δ) from the pre-ischemic spontaneous firing frequency (control) was plotted for each of the 14 individual neurons.

Although the mean spontaneous firing rate shown in Figure 3A appears to increase during ischemia and persist until 30 min after reperfusion, Figure 3B shows that the spontaneous firing rates of individual neurons exhibit several distinct patterns, with considerable variability in both increases and decreases. During ischemia, spontaneous firing increased by ≥0.4 Hz compared with the pre-ischemic control in 10 cases, whereas it decreased by ≤−0.2 Hz in two cases. In these two cases showing a decrease, the reduced firing rate persisted until 120 min after reperfusion. After reperfusion at 5 or 15 min, spontaneous firing increased in four cases (≥1 Hz) compared with the ischemic period.

### 2.4. Changes in Firing Frequency in Response to Mechanical Stimulation

**Figure 4:**
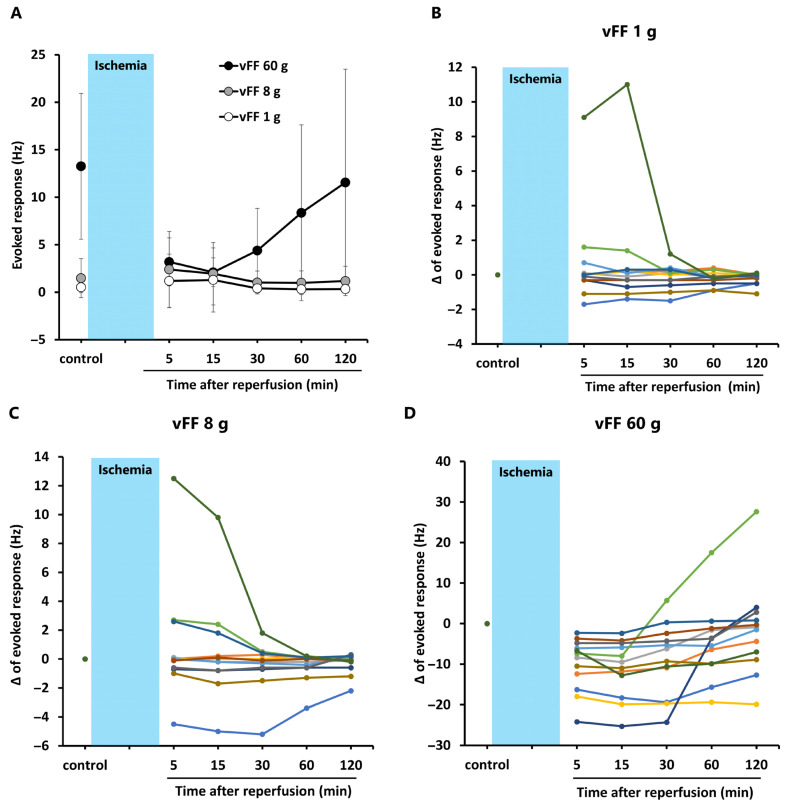
Changes in firing frequency of neurons in the superficial dorsal horn of the rat spinal cord in response to mechanical stimulation after ischemia–reperfusion. The Figure shows how the firing frequency in the superficial dorsal horn changes after ischemia–reperfusion when the rat hindlimb is stimulated with von Frey filaments of 1 g, 8 g, and 60 g. (**A**) Changes in the average firing frequency in response to 1 g, 8 g, and 60 g stimuli. Data are presented as mean ± standard deviation (*n* = 12). (**B**) The change (Δ) from the pre-ischemic firing frequency (control) in response to the 1 g von Frey filament is plotted for each individual neuron. (**C**) Same as in (**B**), but for the 8 g von Frey filament. (**D**) Same as in (**B**), but for the 60 g von Frey filament.

Figure 4 shows how the firing frequency in the superficial dorsal horn changes after ischemia–reperfusion when the rat hindlimb was stimulated with vFFs of 1 g, 8 g, and 60 g. For the average values (Figure 4A), the firing frequency in response to 1 g and 8 g stimuli tended to be higher at 5 and 15 min after reperfusion than pre-ischemia (control), and it returned to baseline by 30 min. However, when looking at individual neuron data (Figure 4B,C), the firing frequency in response to 1 g and 8 g stimuli showed three patterns after ischemia–reperfusion: increased (≥0.7 Hz for both stimuli in three cases), decreased (≤−0.3 Hz for both stimuli in four cases), and little change (five cases). For the 60 g stimulus, the average firing frequency clearly decreased after reperfusion and began to recover from 30 min onward (Figure 4A). Individual data (Figure 4D) showed that firing frequency decreased in all cases 5 and 15 min after reperfusion (≤−2.3 Hz), and this decrease persisted in many cases at 60 and 120 min. In contrast, three cases showed a marked increase in firing frequency after reperfusion, exceeding pre-ischemia levels (≥2.8 Hz at 60 or 120 min).

### 2.5. mRNA Expression Changes in the Spinal Cord

**Figure 5:**
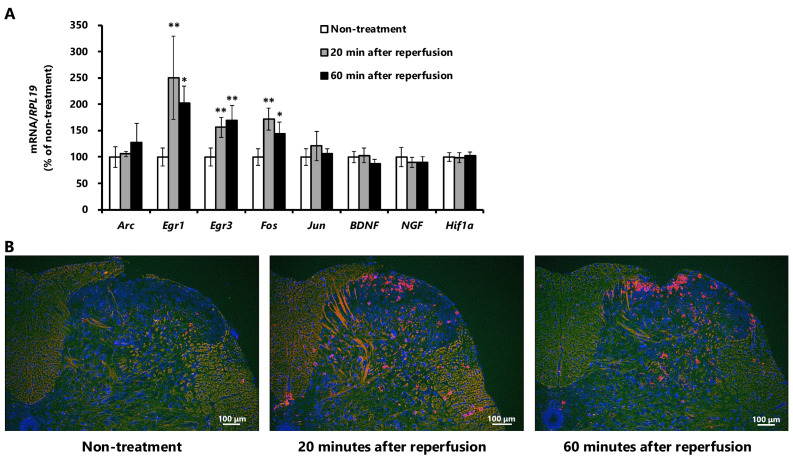
Changes in mRNA expression in the rat spinal cord after ischemia–reperfusion. Spinal cord segments (L4 and L5) were collected from untreated rats and from rats at 20 and 60 min after hindlimb ischemia–reperfusion, and changes in mRNA expression were examined. (**A**) Changes in mRNA expression in the L4 spinal cord of rats, assessed by quantitative RT-PCR. Data are presented as mean ± standard deviation (*n* = 4). ** *p* < 0.01, and * *p* < 0.05 vs. non-treatment (ANOVA with post hoc Dunnett’s test). (**B**) Changes in *Egr1* expression in the superficial dorsal horn of the L5 spinal cord, assessed by in situ hybridization. Red: mRNA signal, and Blue: nuclei.

As shown in Figure 5A, reverse transcriptase-polymerase chain reaction (RT-PCR) analysis showed that the mRNA expression levels of early growth response-1 (*Egr1*), early growth response-3 (*Egr3*), and *Fos* in the L4 and L5 segments of the spinal cord were elevated at 20 and 60 min after reperfusion. Furthermore, in situ hybridization (ISH) analysis (Figure 5B) demonstrated that *Egr1* mRNA was notably upregulated in the superficial dorsal horn at both time points.

## 3. Discussion

Ischemia–reperfusion of the rat hindlimb led to a significant increase in licking behavior, indicating the presence of sensory disturbances. The behavior peaked within the first 10 min after reperfusion, decreasing gradually but persisting throughout the observation period. Plantar blood flow decreased rapidly during ischemia and was almost completely blocked within one minute. Upon reperfusion, blood flow increased markedly and then returned to baseline gradually within about 15 min. These findings are similar to those observed in the mouse ischemia–reperfusion model, suggesting that transient sensory disturbances can also be reproduced in rats.

Extracellular recordings from superficial dorsal horn neurons showed diverse patterns of spontaneous firing and responses to mechanical stimulation. Some neurons showed increased spontaneous firing during ischemia and recovery after reperfusion, whereas others exhibited decreased or unchanged activity. Some neurons showed persistent decreases even after reperfusion. The firing frequency in response to mechanical stimulation (von Frey filaments) also showed variable patterns, with some neurons increasing, decreasing, or showing little change after reperfusion. This diversity highlights the complex neuronal response to ischemia–reperfusion.

Neuropathic sensory symptoms include not only tingling, pricking, pins-and-needles, and pain, but also numbness (decrease or loss of sensation) [18,19], suggesting that both increased and decreased neural activity are involved in a complex neuronal response. In patients with painless diabetic neuropathy, QOL is decreased, and the addition of pain further decreases QOL [24]. In individuals with severe diabetic neuropathy, loss or reduction in sensory function is associated with diminished QOL [25]. It has also been reported that painless numbness affects QOL with greater intensity, leading to further deterioration in QOL [26]. Therefore, the development of therapeutic strategies for sensory disturbances requires an understanding of the reduced neural activity and function that underlie numbness. However, no appropriate evaluation system for this has yet been established. The extracellular recording method from superficial dorsal horn neurons in the ischemia–reperfusion model is considered useful for evaluating neuropathy-related sensory disturbances, since it can simultaneously assess both increased spontaneous firing and hypersensitive responses (thought to result from increased neural activity) and desensitized responses (thought to result from decreased neural activity/function).

Neuronal responses in the superficial dorsal horn to mechanical stimulation of 1 g and 8 g included both hypersensitive and desensitized responses, whereas 60 g mechanical stimulation induced desensitized responses in all cases. It is known that the superficial dorsal horn receives peripheral input from Aδ fibers and C fibers [20], and that C fibers have a higher threshold for activation than Aδ fibers [27,28]. Therefore, the responses to 1 g and 8 g mechanical stimulation appear to be mediated by Aδ fibers, whereas the responses to 60 g mechanical stimulation appear to be mediated by C fibers. Despite the expression of TRPA1 in both C and A fibers [29,30], TRPA1 agonists selectively suppressed monosynaptic excitatory postsynaptic currents evoked by C fibers in the substantia gelatinosa of the spinal cord, but not those evoked by Aδ fibers [31]. These findings raise the possibility that C fibers are more susceptible to desensitization than Aδ fibers.

In the mouse ischemia–reperfusion model, licking behavior was not suppressed by pregabalin, gabapentin, a TRPV1 inhibitor [16], or TRPV1 knockout [17]. Pregabalin and gabapentin are thought to suppress C fiber-mediated responses at the spinal level, and TRPV1 is expressed in C fibers [32,33]. In contrast, both a TRPA1 inhibitor and TRPA1 knockout suppressed licking behavior in the mouse ischemia–reperfusion model [16,17]. Taken together, these findings suggest that licking behavior in the ischemia–reperfusion model involves neural responses mediated by A fibers. In the present study, the increased spontaneous firing and hypersensitivity responses in the spinal dorsal horn recovered between 30 and 60 min after reperfusion, which generally corresponded to the time course of licking behavior. Furthermore, the hypersensitivity response immediately after reperfusion was observed only with weak mechanical stimuli of 1 g and 8 g. Therefore, it is possible that A fibers contribute to the increased spontaneous firing and hypersensitivity responses.

The time course of the desensitized response differed from that of the increases in spontaneous firing and hypersensitivity responses. Most of the desensitized responses persisted even after 60 min of reperfusion. Furthermore, the mRNA levels of the immediate early genes *Egr1* [34], *Egr3* [35], and *Fos* [36] increased in the spinal cord 20 min after ischemia–reperfusion and remained elevated at 60 min after reperfusion. In cardiac systems, Egr1 expression is robustly upregulated following myocardial ischemia–reperfusion and cellular hypoxia/reoxygenation [34]. Suppression of Egr1 reduces inflammation, apoptosis, and infarct size, demonstrating that Egr1 plays a causative role in ischemia–reperfusion-induced injury. These observations suggest that in pathological conditions involving repeated ischemia–reperfusion events, such as diabetic neuropathy and chemotherapy-induced neuropathy, persistent neuronal damage caused by ischemia–reperfusion may accumulate over time. In addition, sustained inflammation and oxidative stress may contribute to the progression and maintenance of this neuronal damage; however, the precise mechanisms involved remain to be clarified.

The primary aim of the present study was to establish an evaluation system for assessing sensory disturbance-related neuronal responses in the rat spinal dorsal horn after hindlimb ischemia–reperfusion. Accordingly, this study was not intended to fully characterize the neuronal subtypes involved, elucidate the underlying pathological mechanisms, including the roles of inflammation and oxidative stress, or examine the pharmacological mechanisms responsible for the hypersensitive and desensitized response patterns. These issues should be addressed in future studies, together with analyses using models of diabetic neuropathy and chemotherapy-induced neuropathy, to identify differences in neural responses compared with the ischemia–reperfusion model and to further clarify the contribution of the spinal cord to altered sensory perception.

This study demonstrated that ischemia–reperfusion of the rat hindlimb induces sensory disturbances, as evidenced by increased licking behavior and diverse neuronal responses in the spinal dorsal horn. Both hypersensitive and desensitized responses were observed, reflecting the complex sensory changes induced by ischemia–reperfusion. Importantly, this ischemia–reperfusion model provides valuable insights into the mechanisms underlying sensory disturbances observed in diseases such as diabetic neuropathy and chemotherapy-induced neuropathy. By analyzing neural responses in this model, it is possible to gain a deeper understanding of the transmission and regulation of sensory disturbances, and by using desensitized responses as indicators of nerve damage, this approach is expected to contribute to the development of new treatments and drugs.

## 4. Materials and Methods

### 4.1. Animals

Male Sprague Dawley rats (7 weeks old) obtained from Japan SLC, Inc. (Shizuoka, Japan), or Charles River Laboratories Japan (Yokohama, Japan) were used in the experiments. All animals were housed in a room under controlled conditions (temperature 22–23 °C; relative humidity 45–65%; 12 h light/dark cycle) and given ad libitum access to food and water. All animal procedures were approved by the Committee for Animal Experiments at the University of Toyama or the Institutional Animal Care Committee of the Pharmaceutical Research Center of Asahi Kasei Pharma Corporation.

### 4.2. Ischemia–Reperfusion of the Hind Paw

To induce hind paw ischemia, a glabrous region just proximal to the ankle joint was firmly compressed with a cut rubber band (Oband, no. 16; Kyowa Limited, Osaka, Japan) for 5–60 min. Reperfusion was achieved by cutting the rubber tourniquet. The ischemia–reperfusion procedure for the measurement of licking behavior was performed in unanesthetized rats. In contrast, blood flow assessment, in vivo extracellular recordings, and spinal cord sampling were conducted under anesthesia (see below).

### 4.3. Measurement of Hind Paw Blood Flow

Rats were anesthetized with isoflurane. A laser Doppler flowmeter (ALF21; Advance Co., Ltd., Tokyo, Japan) was used to assess blood flow in the plantar skin of the hind paw. The probe (Type N, diameter 0.5 mm) was held 1 mm from the plantar surface to avoid mechanical and thermal effects on blood flow. Flux signals were digitized through an analog-to-digital converter (Digidata 1400A; Molecular Devices, Union City, CA, USA) and stored on a personal computer using Clampex software (version 10.2; Molecular Devices, San Jose, CA, USA) for data acquisition and Clampfit software (version 10.2; Molecular Devices, San Jose, CA, USA) for data analysis.

### 4.4. Measurement of Licking Time After Ischemia–Reperfusion

Rats were placed individually in observation chambers with an acrylic transparent floor. After a 30–45 min acclimation period, the rats underwent compression ischemia for 5–60 min as described above. Immediately after cutting the rubber tourniquet, the rats were placed back into their chambers, and their behaviors were videotaped with no one present. The time spent licking the treated hind paw was measured with a stopwatch during video playback.

### 4.5. In Vivo Extracellular Recordings of the Spinal Cord Dorsal Horn

In vivo extracellular recordings of the spinal cord dorsal horn were performed as previously described [22,23]. Briefly, after administering urethane (1.2–1.5 g/kg, i.p.) to anesthetize the rat, the spine was exposed over 6 to 7 vertebral segments from the thoracic to the sacral vertebrae, and the lamina was removed to avoid damaging the spinal cord. The rats were then placed in a stereotaxic instrument. The dura mater was removed under a microscope, and the spinal cord surface was exposed by oxygen loading with carbon dioxide (95% O_2_, 5% CO_2_) and 37 ± 1 °C warmed Krebs solution (117 mM NaCl, 3.6 mM KCl, 2.5 mM CaCl_2_, 1.2 mM MgCl_2_, 1.2 mM NaH_2_PO_4_, 11 mM glucose, and 25 mM NaHCO_3_) at 37 °C, while perfusing the surface of the spinal cord at 10–15 mL/min. A tungsten microelectrode (tip diameter: 25 µm; tip impedance: 9–12 MΩ; FHC, Bowdoin, ME, USA) was inserted. Spinal cord dorsal horn neuron recordings were made from a range of 20–150 µm from the surface corresponding to the I–II layers. ClampFit software package (version 10.2, Molecular Devices) or Sutter patch software (version 2.3.1, Sutter Instrument, Novato, CA, USA) was used to amplify (EX1, Dagan Corporation, Minneapolis, MN, USA), digitize (Digidata 1550A, Molecular Devices, or IPA, Sutter Instrument), and display unit signals online. Next, the areas of the skin that would produce a nerve response when pinched with forceps or touched with a cotton swab or light brush were identified. Recordings were made only from the spinal cord dorsal horn neurons in the area from the ankle to the toes, where blood flow changes occurred due to the ischemia–reperfusion procedure. We searched for areas on the skin where neurons responded to a light brush touch or pinching with tweezers. Mechanical stimuli were applied to the skin of the ipsilateral hind limb using vFFs (North Coast Medical, Morgan Hill, CA, USA) with a bending force of 1–60 g at the maximal response point within each neuron’s receptive area. Mechanical stimulation was then applied for 10 s to the previously identified maximal response point immediately before the ischemic procedure and at 5, 15, 30, 60, and 120 min after reperfusion during recording.

### 4.6. Spinal Cord Sampling

Under isoflurane anesthesia, total blood was collected from the abdominal aorta, followed by exsanguination and euthanasia. The L4 spinal cord (for quantitative RT-PCR) and L5 spinal cord (for ISH) were extracted. The L4 spinal cord was fixed in neutral buffered formalin solution, and the L5 spinal cord was fixed in RNAlater (AM7021; Thermo Fisher Scientific Inc., Waltham, MA, USA).

### 4.7. Quantitative RT-PCR

Spinal cord tissue pieces were immersed in 1 mL of RNAlater. After storage at 4 °C for one day, the tissue was removed and stored at −20 °C until RNA extraction. RNA was extracted using the RNeasy Lipid Tissue Mini Kit (74804; QIAGEN, Hilden, Germany) according to the manufacturer’s protocol. Then, cDNA synthesis was performed using the SuperScript VILO cDNA Synthesis Kit (11754-050; Thermo Fisher Scientific Inc.) according to the manufacturer’s protocol, and qPCR was performed using the THUNDERBIRD Probe qPCR Mix (QPS-101; TOYOBO CO, LTD, Osaka, Japan) according to the manufacturer’s protocol. Quantitative RT-PCR was conducted using the TaqMan method. Target genes were amplified by using specific primers for *Egr1* (forward: 5′-CGAACAACCCTACGAGCAC-3′; reverse: 5′-GCGCCTTCTCGTTATTCAGA-3′; probe: Universal Probe No. 114 (Cat. No. 04693485001, Roche, Mannheim, Germany)), *Egr3* (forward: 5′-CGGTAGCCCATTACACTCAGA-3′; reverse: 5′-AGTAGGTCACGGTCTTGTTGC-3′; probe: Universal Probe No. 20 (Cat. No. 04686934001, Roche)), *Fos* (forward: 5′-GCTCCCCTGTCAACACACA-3′; reverse: 5′-GACCAGAGTGGGCTGCAC-3′; probe: Universal Probe No. 22′ (Cat. No. 04686969001, Roche)), *Arc* (forward: 5′-CTCAGGGTGAGCTGAAGCA-3′; reverse: 5′-TCTTCACTGGTATGAATCACTGC-3′; probe: Universal Probe No. 79 (Cat. No. 04689020001, Roche, Basel, Switzerland)), *Jun* (forward: 5′-GCCACCGAGACCGTAAAG-3′; reverse: 5′-CTGTGCGAGCTGGTATGAGT-3′; probe: Universal Probe No. 7 (Cat. No. 04685059001, Roche)), *BDNF* (forward: 5′-GTGGAGGCTAAGTGGAGCTG-3′; reverse: 5′-CAGGATGGCCACTCAGAAAT-3′; probe: Universal Probe No. 15 (Cat. No. 04685148001, Roche)), *NGF* (forward: 5′- GTGTCAGTGTGTGGGTTGGA-3′; reverse: 5′-TCACCTCCTTGCCCTTGAT-3′; probe: Universal Probe No. 114 (Cat. No. 04693485001, Roche)), *HiF1a* (forward: 5′-CATGATGGCTCCCTTTTTCA-3′; reverse: 5′-CATAGTAGGGGCACGGTCAC-3′; probe: Universal Probe No. 18 (Cat. No. 04686918001, Roche)), and *RPL19* (forward: 5′-GACCCCAATGAAACCAACGA-3′; Reverse: 5′-TCAGGCCATCTTTGATCAGCTT-3′; Probe: 5′-FAM-CGCCAATGCCAACTCTCGTCAACAG-TAMRA-3′). QuantStudio 7 Flex Real-Time PCR System (Thermo Fisher Scientific Inc.) was used for Quantitative RT-PCR measurement. *RPL19* was used as the housekeeping mRNA for normalization. The mRNA amount in untreated mice was set to 100%, and the expression levels at 20 min and 60 min after reperfusion were calculated.

### 4.8. In Situ Hybridization (ISH)

Tissue was fixed by immersion in neutral buffered formalin solution for at least one day, and 2 µm-thick paraffin sections were prepared. ISH was performed using the QuantiGene ViewRNA ISH Tissue Assay Kit (QVT0400, QVT0410; Thermo Fisher Scientific Inc.) according to the manufacturer’s protocol. *Egr1* ISH probe (VC1-20417; Thermo Fisher Scientific Inc.) was used. Stained images were captured using a fluorescence microscope (BZ-9000, KEYENCE CORPORATION, Osaka, Japan), mRNA was detected by Fast Red fluorescence, and nuclei were detected by Hoechst blue fluorescence. To visualize tissue contours, images were also taken with green fluorescence unrelated to the signal, and red, blue, and green fluorescence images were merged.

### 4.9. Statistical Analysis

All measured values were expressed as the mean  ±  standard deviation. The one-way ANOVA followed by post hoc Dunnett’s test was used for all statistical testing, and SAS (ver. 9.4; SAS Institute, Cary, NC, USA) and EXSUS (ver. 10.0; CAC EXICARE, Tokyo, Japan) were used for all statistical analyses. For all tests, values of *p*  <  0.05 were considered statistically significant.

## Data Availability

The data presented in this study are available on request from the corresponding author.

## Abbreviations

The following abbreviations are used in this manuscript:

QOL	Quality of life
vFF	von Frey filament
RT-PCR	Reverse transcriptase–polymerase chain reaction
ISH	In situ hybridization
ANOVA	Analysis of variance
